# Long-Term Green Tea Supplementation Does Not Change the Human Gut Microbiota

**DOI:** 10.1371/journal.pone.0153134

**Published:** 2016-04-07

**Authors:** Pilou L. H. R. Janssens, John Penders, Rick Hursel, Andries E. Budding, Paul H. M. Savelkoul, Margriet S. Westerterp-Plantenga

**Affiliations:** 1 Department of Human Biology, School for Nutrition and Translational Research in Metabolism (NUTRIM), Maastricht University, Maastricht, The Netherlands; 2 Department of Medical Microbiology, School for Nutrition and Translational Research in Metabolism (NUTRIM), Maastricht University Medical Center+, Maastricht, the Netherlands; 3 Department of Medical Microbiology & Infection Control, VU University Medical Center, Amsterdam, The Netherlands; GI Lab, UNITED STATES

## Abstract

**Background:**

Green tea catechins may play a role in body weight regulation through interactions with the gut microbiota.

**Aim:**

We examined whether green tea supplementation for 12 weeks induces changes in composition of the human gut microbiota.

**Methods:**

58 Caucasian men and women were included in a randomized, placebo-controlled design. For 12 weeks, subjects consumed either green tea (>0.56 g/d epigallocatechin-gallate + 0.28 ∼ 0.45 g/d caffeine) or placebo capsules. Fecal samples were collected twice (baseline, vs. week 12) for analyses of total bacterial profiles by means of IS-profiling, a 16S-23S interspacer region-based profiling method.

**Results:**

No significant changes between baseline and week 12 in subjects receiving green tea or placebo capsules, and no significant interactions between treatment (green tea or placebo) and time (baseline and week 12) were observed for body composition. Analysis of the fecal samples in subjects receiving green tea and placebo showed similar bacterial diversity and community structures, indicating there were no significant changes in bacterial diversity between baseline and week 12 in subjects receiving green tea capsules or in subjects receiving placebo capsules. No significant interactions were observed between treatment (green tea or placebo) and time (baseline and week 12) for the gut microbial diversity. Although, there were no significant differences between normal weight and overweight subjects in response to green tea, we did observe a reduced bacterial alpha diversity in overweight as compared to normal weight subjects (p = 0.002).

**Conclusion:**

Green tea supplementation for 12 weeks did not have a significant effect on composition of the gut microbiota.

**Trial Registration:**

ClinicalTrials.gov NCT01556321

## Introduction

Green tea (GT) catechins have been shown to have anti-obesity effects in humans. In particular a decrease in body weight [1–5] and body fat loss [1, 3, 4] has been reported in response to GT catechins consumption. Potential underlying mechanisms include the increase in energy expenditure, thereby preserving fat free body mass, and the promotion of fat oxidation [6–10]. In addition a reduced fat absorption has been suggested [11], although evidence for long-term effects is lacking [12]. Moreover, a role of gut microbiota has been suggested, possibly in relation to the preservation of fat free body-mass and the increase in energy expenditure [13].

The human intestinal microbiota constitutes a complex ecosystem, in which the majority of bacterial species belong to four phyla: *Firmicutes*, *Bacteroidetes*, *Proteobacteria* and *Actinobacteria*. The bacterial composition is rather stable within adults, whereas there is a large inter-individual variation in bacterial composition, as each individual has its own personal microbial community [14]. Over the past few years animal studies as well as human studies have suggested a role of the indigenous microbiota in body weight regulation [15]. Body fat of germ-free mice increased after gut microbiota transplantation from normal mice, without an increase in food consumption [16], suggesting that the gut microbiota is important in regulation of energy harvesting and fat storage. Moreover, transplantation of the gut microbiota from obese mice into lean germ-free mice led to greater fat deposition than transplants from lean donors [17]. Likewise, when fecal microbiota from obese and lean people was transplanted into germ-free mice, body weight only increased in mice receiving microbiota from obese people [18]. Thus, the microbiota in the intestines of obese individuals may be more efficient in extracting energy from the diet and in storing this energy as fat, resulting in an increase in body weight and body fat percentage. Besides that, gut microbiota may also affect body weight regulation via metabolic signalling of short chain fatty acids through G-protein coupled receptors, to regulate appetite hormones and modulate inflammation [19]. Several studies have actually found a difference in composition of the gut microbiota between normal weight and obese people [15, 20, 21], and have shown that bacterial diversity is reduced in obese compared with normal weight people [22, 23].

Although the composition of the human gut microbiota is considered to be essentially stable in adults, changes in diet may influence bacterial composition [24]. Polyphenols for example, have a weight lowering effect [4, 5, 25], which is thought to be related to a change in the gut microbiota [13]. The gut microbiota is able is to metabolize a part of the polyphenols [26, 27], moreover the cleavage of glycosidic linkages in polyphenols generates glycans that are important as a nutrient foundation for the gut microbiota [28] especially for the *Bacteroidetes*, as they are supposed to have a higher glycan degrading capacity than *Firmicutes*. Therefore polyphenols may alter the balance between these two phyla in favour of *Bacteroidetes*. Furthermore, polyphenols may have a prebiotic effect as Rastmanech et al showed a weight lowering effect of polyphenols in obese subjects [13]. The prebiotic effects of GT catechins on human gut microbiota are still poorly understood; only a few studies have investigated the effect of catechins on the gut microbiota. Most of these studies found that catechin inhibits growth of certain pathogenic bacteria, including *Clostridium difficile [29]* and *Staphylococcus spp*. [30, 31], *w*hile two studies found that catechins stimulate growth of beneficial commensal bacteria *Bifidobacterium spp*. [30, 32, 33]. However, the long-term effect of catechins on energy extraction via possible changes in gut bacterial community structure is still unknown. Therefore, we investigated whether green tea induced changes in composition and diversity of the human gut microbiota. Furthermore we investigated whether there was a difference in composition of the gut microbiota between normal weight and overweight subjects at these levels and their possible differences in response to green tea.

## Subjects and Methods

Subjects were recruited by advertisements on notice boards at Maastricht University and in local newspapers. Subjects were healthy, normal weight (Body Mass Index (BMI) 18–25 kg/m^2^) or overweight/obese (BMI >25 kg/m^2^) Caucasians, aged between 18–50 yrs were included in the study. All subjects were non-smoking, healthy, weight stable (weight change < 3kg during the past 6 months), not consuming a more than moderate amount of alcohol (<10 consumptions/week) and caffeine (<100 mg/day). Subjects were not using antibiotics during the last 6 months and were free of medication except for use of oral contraceptives in women. Furthermore subjects with Crohn's disease, ulcerative colitis or diverticulitis were excluded. Pregnant and lactating women were also excluded from participation.

Subject sample size was calculated using total fecal fat excretion data from a previous study to calculate the effect size [11], as this was also a primary endpoint of the study. Fecal fat excretion data were published elsewhere [34]. Sample size was calculated as 25 subjects per group. Taking a drop-out into account, the sample size was finalized as 60 subjects. A written informed consent was obtained from all the participants. This study was conducted according to the guidelines laid down in the Declaration of Helsinki and the Medical Ethics Committee of the Academic Hospital in Maastricht approved the study.

### Study protocol

The study was conducted in a randomized, single blind, placebo-controlled design with two randomly sequenced experimental groups: GT or placebo (PL). The green tea and placebo groups were stratified (pre-stratification) according to the number of men and women, BMI and age. Subjects visited the university twice (at baseline, and after 12 weeks). They arrived at fasted state at 08:30h and voided their bladder before anthropometric measurements. After baseline measurements, subjects received either GT or PL capsules, which they had to consume daily for a period of six weeks. Six weeks after baseline, subjects received an additional supply of capsules containing the same treatment, which they had to consume daily for the last six weeks of the intervention. They were instructed to abstain from GT and to use less than 100 ml caffeine-containing beverages per day. Subjects were asked to maintain their food intake pattern. During the four consecutive days before baseline they were asked to record all the food and drinks they consumed, in order to be able to consume exactly the same during the four days before their final measurements at week 12. They were instructed again to have the same diet during the four consecutive days before their visit in week 12 according to the recorded days before the baseline measurement **(S1 Table).** Subjects had to maintain their habitual activity level.

### Dosage

After baseline measurements, subjects received either capsules with GT extract (containing > 0.06 g Epigallocatechin-3-gallate (EGCG) and 0.03 ∼ 0.05 g caffeine per capsule) or PL capsules (**Table 1**), which they consumed daily for 12 weeks. They had to consume nine capsules per day: three capsules between breakfast and lunch, three capsules between lunch and dinner, and three capsules in more than 2 hours after dinner. Subjects were instructed not to consume the capsules simultaneously with their meals in order to prevent confounding effects of the macronutrients [35]. GT extract (Sunphenon 70H-T) was manufactured by Taiyo kagaku Co Ltd., Mie, Japan. Dutch BioFarmaceutics B.V encapsulated GT and PL components. The capsules with GT are intended for use as a dietary supplement. When specifying the PL capsules the GT extract was replaced with microcrystalline cellulose (0.27 g per capsule in the GT capsules vs. 0.38 g in the PL capsules). Microcrystalline cellulose is refined wood pulp, and often used in vitamin supplement. The capsules all had the same appearance. Compliance of capsule intake was checked by asking subjects to return all remaining capsules after 6 and 12 weeks. Bioactivity of the GT capsules was tested previously, in a randomized crossover experiment by Hursel *et al*. [35] in which comparable GT from Taiyo (Sunphenon) was used.

**Table 1:** Composition of the green tea extract and placebo capsules and total dose consumed per day.

	Green tea capsules		Placebo capsules	
	per capsule	per day	per capsule	per day
**Capsules**				
Total weight, g	0.65	5.82	0.51	4.60
Weight capsule, g	0.12	1.07	0.12	1.07
Weight filling, g	0.53	4.76	0.39	3.53
**Filing**				
Microcrystalline cellulose, g	0.27	2.43	0.38	3.44
Colloidal silicon dioxide. mg	5.50	49.5	7.80	70.2
Magnesium stearate, mg	2.60	23.4	2.60	23.4
Active filling, g	0.25	2.25		
**Active filling**				
Caffeine, g	0.03 ∼ 0.05	0.28 ∼ 0.45		
Polyphenols, g	0.20 ∼ 0.22	1.80 ∼ 1.97		
**Polyphenols**				
Catechins, g	> 0.15	> 1.35		
Other polyphenols, g	< 0.07	< 0.62		
**Catechins**				
Epigallocatechin gallate, g	> 0.06	> 0.56		

Green tea extract: Sunphenon 70H-T (Taiyo Kagaku Co. Ltd., Mie, Japan). Dutch BioFarmaceutics B.V encapsulated green tea and placebo components. Microcrystalline cellulose was used as filler, colloidal silicon dioxide and magnesium stearate were used as anti-caking agent. Capsules were made of hydroxypropyl methylcellulose. Titanium dioxide and copper complexes of chlorophyll were used as food colouring. Subjects consumed nine capsules per day.

### Body composition

Body weight was measured at baseline and after 12 weeks using a digital balance and height by a wall-mounted stadiometer. BMI was calculated as body weight (kg) divided by height (m) squared. Fat mass was determined by Bod Pod (Life measurement, Inc) measurements (air displacement plethysmography) [36]. Fat mass index (FMI) was calculated by fat mass (kg) divided by height (m) squared. BMI, % body fat and FMI were used to define body composition. Waist and hip circumferences were determined in standing position by a tape measure. Waist circumference was measured at the smallest circumference between rib cage and iliac crest, and hip circumference at the level of the spina iliaca anterior superior. Accordingly, waist-to-hip ratio (WHR) was calculated by dividing waist by hip circumference. WHR was used to define body fat distribution.

### Microbiota

One day before the baseline visit and the visit in week 12, fecal samples were collected in pre-weighed plastic containers for analyses of the gut microbiota. Fecal samples were collected by subjects at home, delivered at the University within 24h, and subsequently stored at -80°C until further processing.

#### DNA isolation

DNA was extracted from fecal samples with the easyMAG extraction kit according to the manufacturer's instructions (Biomérieux, Marcy l’Etoile, France). 100-400mg of feces was placed in an Eppendorf tube with 200μl of nucliSENS lysis buffer, and vortexed. Tubes were incubated shaking for 5 minutes at room temperature. After centrifugation (13000 rpm; 2 min), 100 μl of the supernatant was transferred to an easyMAG isolation container containing 2 ml of nucliSENS lysis buffer. This suspension was incubated for 10 min at room temperature after which 70 μl of magnetic silica beads were added. The easyMAG automated DNA isolation machine was used following the “specific A” protocol, eluting DNA in 110 μl buffer and stored at 4°C until further analysis.

#### IS-profiling of the intestinal microbiota

For IS profiling, DNA samples were diluted 1:10. Amplification of IS-regions was performed with the IS-pro assay (IS-diagnostics, Amsterdam, the Netherlands) according to the protocol provided by the manufacturer. IS-pro is a validated technique that combines differentiation bacterial species by the length of the 16S–23S rDNA intergenic spacer (IS) region with taxonomic classification by phylum-specific fluorescently labelled PCR primers [37]. The procedure consists of two multiplex PCRs: the first PCR contains two different fluorescently labeled primers. One amplifying the phyla *Firmicutes*, *Actinobacteria*, *Fusobacteria* and *Verrucomicrobia (FAFV*) and the other labeled primer for the phylum *Bacteroidetes*. A separate PCR with a third labeled primer is performed for the phylum *Proteobacteria*. Amplifications were carried out on a GeneAmp PCR system9700 (Applied Biosystems, Foster City, CA). After PCR, 5μl of PCR product was mixed with 20μl formamide and 0.2μl custom size marker (IS-diagnostics). Fragment analysis of the DNA was performed on an ABI Prism 3500 Genetic Analyzer (Applied Biosystems). IS-profiling resulted in microbial profiles. Each profile consisted of a set of color-labelled peaks; with each peak related to a specific IS fragment (measured in nucleotides) and the color related to a specific phylum group (*FAFV*, *Bacteroidetes* or *Proteobacteria*).

### Data analysis

For data-visualisation of the bacterial composition, the Spotfire software package (TIBCO, Palo Alto, CA, USA) was used. All intensities were log2 transformed; this log2 transformation compacts the range of variation in peak heights, reduces the dominance of high peaks and includes less abundant species in downstream analyses. This conversion was used in all downstream analyses, e.g. calculating between-sample microbial diversity. Microbial diversity was calculated both per phylum and for the overall microbial composition (by pooling all phyla together) using R package version “[38](“R package 3.1.3”)”.

#### Alpha diversity and microbial profiles

Alpha diversity describes the within-sample bacterial diversity and was used to examine the effect of green tea on the microbial diversity. Alpha-diversity metrics calculate the microbial diversity for each sample separately. To quantify and compare the alpha diversity the Shannon index was calculated using the R package vegan “[39](“R package 2.3–0”)”. The Shannon diversity index is a diversity index that accounts for species abundance as well as the evenness of the species present. A heat map was created by generating a correlation matrix of the log2 transformed profile data and hierarchical clustering of samples by the unweighted pair group method with arithmetic mean (UPGMA).

#### Beta diversity

Beta diversity captures the dissimilarity in bacterial composition between samples to examine the changes in the overall microbial community structure during the intervention period. Between-sample diversity, was analyzed by comparing the community structures, using the Bray-Curtis distance, a commonly used metric in microbial ecology [40]. Bray-Curtis dissimilarities were calculated, using the R package vegan, to indicate the changes in the microbiota over the course of the intervention period. The Bray Curtis-dissimilarity indicates the distance between two samples, and as such the distance between the baseline sample and the follow-up sample could be calculated for each individual seperately. As such, one value was constructed to indicate the change in the microbiota over time. These data were subsequently used to compare the change in microbiota community structure in the green tea group as compared to the placebo group (i.e. to test whether the Bray Curtis-distance was significantly larger in the intervention group). Between-sample diversity was visualized by principal coordinates analysis (PCoA).

#### Statistical analysis

The Statistical Package for the Social Sciences 20.0 (SPSS) was used to test the effects of green tea on body composition, microbial diversity and community structures and to test the differences in microbial diversity between normal weight and overweight subjects. The green tea and placebo groups were stratified (pre-stratification) according to the number of men and women, BMI and age, and the differences between these groups were checked with the use of a factorial ANOVA. P values for these differences were >0.90 for sex distribution and BMI, >0.80 for age, and >0.50 for body weight. All statistical tests were two-sided and differences were considered statistical significant if p<0.05. Values are expressed as means and standard deviations unless otherwise stated.

Within our study we examined the following objectives:

1 To examine the effects of green tea on gut microbial diversity (alpha diversity), body weight and body composition

For these analyses the data on microbial diversity, body composition and body weight at baseline as well as week 12 from subjects in both the green tea and placebo group were included. A paired samples t-test was used to determine whether the gut microbial diversity, body composition and body weight changed over time (between baseline and week 12) in subjects receiving green tea and in subjects receiving placebo. A mixed analysis of variance (ANOVA) was used to determine whether any change in gut microbial diversity, body composition and body weight is the result of the interaction between the "type of treatment" (i.e., green tea or placebo, which is our between-subjects factor) and "time" (i.e., baseline or week 12, which is our within-subjects factor). Data were normally distributed; this was tested using the Shapiro-Wilks test.

2 To examine the changes in the overall microbial community structure (beta diversity) between baseline and week 12 in the green tea group as compared to the placebo group.

The change in the microbial community structure between baseline and samples at week 12 within each individual was calculated using the Bray-Curtis dissimilarity.

This implies that for each individual one value was calculated for the distance between baseline and follow-up (week 12) sample. As these distances were not normally distributed, a Mann Whitney test, the non-parametric equivalent of the independent t-test was used to examine whether the dissimilarity in microbial community structures (Bray-Curtis distance) was significantly different in the green tea group as compared to the placebo group.

3 To examine the differences in microbial (alpha) diversity between normal weight and overweight subjects and their possible differences in effects of green tea on microbial diversity, body weight and body composition.

A mixed ANOVA design was used to investigate the effects of green tea on body weight, body composition and bacterial diversity between normal weight and overweight subjects. Subjects were divided in BMI categories; normal weight (NW): BMI 18–25 kg/m^2^ and overweight (OW): BMI > 25 kg/m^2^ and two treatment conditions; green tea (GT) and placebo (PL), which resulted in four subgroups (GT/NW, GT/OW, PL/NW, PL/OW). To determine whether there was a difference in composition of the bacterial alpha diversity between normal weight and overweight subjects (no treatment effect), Mann-Whitney test was used. Comparisons of the microbial diversity of normal weight and overweight subjects were performed in a non-parametric fashion, as the data between subjects were not normally distributed. For the latter, only baseline samples were used.

## Results

### Subjects

**Fig 1:**
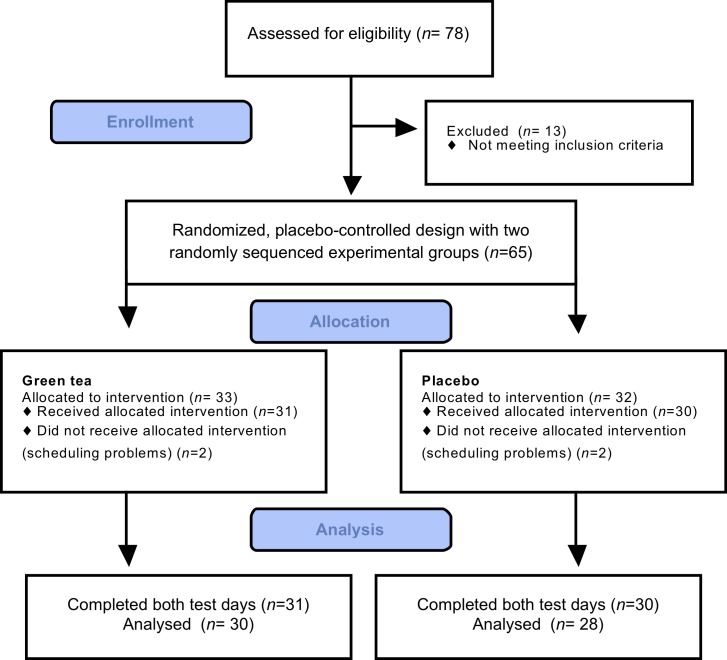
CONSORT flow diagram.

In this study, 65 subjects started the experiments; four subjects dropped out due to scheduling problems and samples of three subjects were not included due to incomplete IS-profiles at baseline (one subject) or week 12 (two subjects) **(Fig 1)**. The final sample size was 58 subjects (46 women, 12 men). Groups were not significantly different at baseline with respect to age, sex and anthropometry **(Table 2).** Data showed no significant changes in body composition between baseline and week 12 in subjects receiving green tea capsules or in subjects receiving placebo capsules. No significant interactions were observed between treatment (green tea or placebo) and time (baseline and week 12) for body composition. Mean reported energy and fat intakes did not significantly differ between groups. Results on effects of GT on body composition and data on reported intake were published elsewhere [34]. Furthermore, there were no significant differences in the response to GT between men and women. Compliance was checked from counting the left-over and returned capsules. Subjects in the GT group returned 2.9±1.8 capsules/week and subjects in the PL group returned 3.0±1.7 capsules/week, without a significant difference in returned capsules between both groups.

**Table 2:** Subject characteristics at baseline and week 12 in the green tea and placebo groups (n = 58).

	Green tea						Placebo					
	Men (*n* = 6)		Women (*n* = 24)		Total (*n* = 30)		Men (*n* = 6)		Women (*n* = 22)		Total (*n* = 28)	
	Baseline	Week 12	Baseline	Week 12	Baseline	Week 12	Baseline	Week 12	Baseline	Week 12	Baseline	Week 12
Age, years	28.9±10.4	-	28.2±10.8	-	28.2±10.8	-	29.1±10.9	-	28.7±10.2	-	28.1±10.5	-
Height, m	1.71±0.09	-	1.70±0.08	-	1.70±0.08	-	1.70±0.10	-	1.70±0.07	-	1.70±0.09	-
Body weight, kg	68.9±14.6	68.9±14.7	66.8±14.1	66.7±14.2	66.8±14.1	66.7±14.2	69.2±14.3	69.3±14.9	63.2±9.0	63.1±9.4	67.5±14.0	67.8±14.5
BMI, kg/m^2^	23.6±4.2	23.6±4.3	23.0±4.0	23.0±4.0	23.0±4.0	23.0±4.0	24.0±4.8	24.1±5.0	22.9±3.9	22.9±4.1	23.6±4.6	23.7±4.8
FMI, kg/m^2^	7.1±3.4	7.1±3.5	6.9±3.1	6.9±3.2	6.9±3.1	6.9±3.2	7.5±3.7	7.6±3.8	7.3±3.3	7.5±3.4	7.2±3.5	7.4±3.6
FFMI, kg/m^2^	16.5±2.0	16.5±1.8	16.1±1.9	16.1±1.8	16.1±1.9	16.1±1.8	16.5±2.1	16.5±2.3	15.6±1.2	15.5±1.2	16.3±2.0	16.3±2.2
WHR	0.76±0.10	0.76±0.08	0.76±0.09	0.76±0.08	0.76±0.09	0.76±0.08	0.74±0.09	0.74±0.09	0.70±0.04	0.70±0.05	0.73±0.08	0.74±0.09
FM, kg	20.6±9.6	20.6±10.1	19.9±8.9	19.9±9.2	19.9±8.9	19.9±9.3	21.3±9.4	21.5±9.6	19.9±7.8	20.3±8.3	20.4±9.0	20.7±9.3
FFM, kg	48.4±9.4	48.3±9.0	46.9±9.1	46.8±8.7	46.9±9.1	46.8±8.7	48.0±9.6	47.8±10.0	43.3±4.6	42.8±4.4	47.2±9.1	47.1±9.5
Body fat, %	29.1±9.0	29.0±9.4	29.1±8.2	29.1±8.5	29.1±8.2	29.1±8.5	30.1±9.3	30.3±9.5	30.7±8.2	31.2±8.5	29.5±8.7	29.7±9.0

BMI: Body mass index; FMI: Fat mass index; FFMI: Fat free mass index; WHR: Waist-to-hip ratio; FM: Fat mass; FFM: Fat free mass. Values are means ± standard deviations. Data were analyzed by a mixed ANOVA. Groups did not differ significantly at baseline.

### Diversity analysis

**Fig 2:**
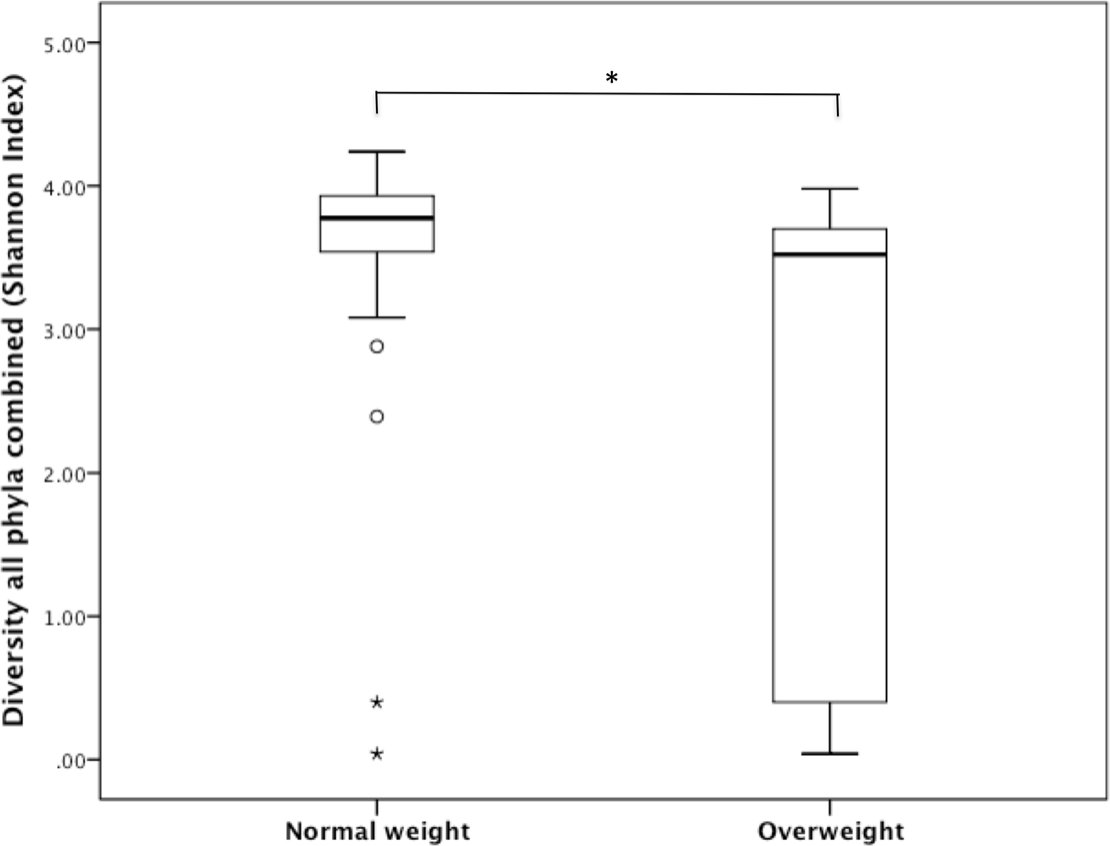
BMI categories: BMI 18–25 kg/m^2^ (*Mdn* = 3.78, *n* = 43) vs. BMI ≥25 kg/m^2^ (*Mdn* = 3.52, *n* = 17). All Phyla combined, Shannon diversity index using Mann-Whitney test, *p = 0.002.

There was no significant difference between normal weight (BMI 18–25 kg/m^2^) and overweight (BMI >25 kg/m^2^) subjects in their response to GT. However, when baseline samples were compared, the Shannon diversity index indicated that the (alpha) diversity for all phyla combined was significantly lower in overweight subjects (*Mdn* = 3.52, *n* = 17) compared with normal weight (*Mdn* = 3.78, *n* = 43) subjects (*U* = 180, *r* = -0.39, p = 0.002, **Fig 2**).

**Fig 3:**
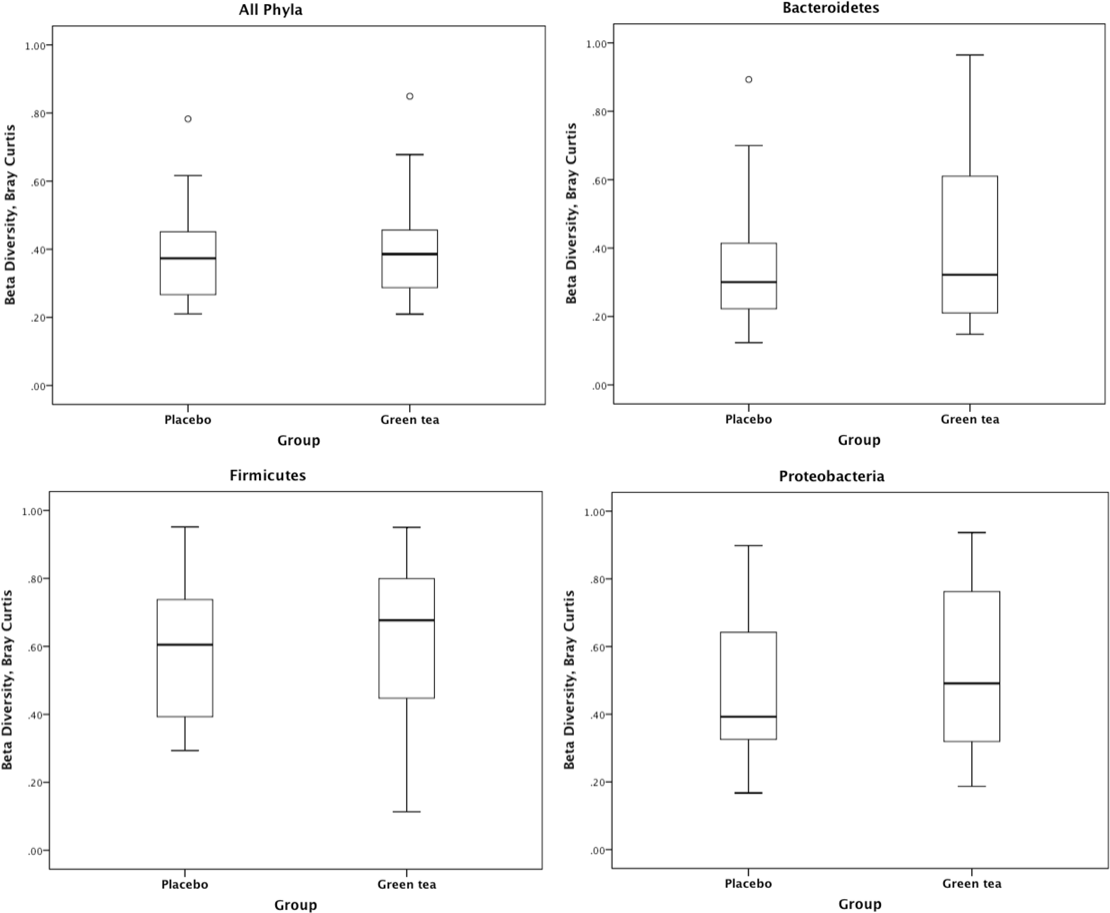
Box plots, comparisons of the between-sample diversity in green tea and placebo as calculated by Bray Curtis dissimilarity. Beta diversity captures the dissimilarity in microbial composition of the groups between baseline and week 12.

Analysis of the fecal samples in subjects receiving GT and PL showed that the alpha diversity for all phyla combined as well as the diversity of *Bacteroidetes*, *FAVF* and *Proteobacteria* did not significantly change between baseline and week 12 in subjects receiving green tea capsules nor in subjects receiving placebo capsules. No significant interactions were observed between treatment (green tea or placebo) and time (baseline and week 12) for alpha diversity of all phyla combined, *Bacteroidetes*, *FAVF* and *Proteobacteria* (**Table 3**). A heat map was generated from all IS-profiles stratified by phyla. Absence of clustering was displayed in this heatmap as there was no separation by group and/or week (**S1 Fig**)**.** Bray-Curtis index was used to assess the (dis)similarity in the microbial community structure between samples, the average Bray-Curtis dissimilarity (all phyla combined) of paired baseline and week 12 samples was not significantly different between GT and PL groups (**Fig 3**). This indicates that the (potential) change in microbiota composition during the intervention period was not significantly larger in subjects receiving GT as compared to PL.

**Table 3:** Mean bacterial diversity per phylum in the green tea and placebo groups at baseline and week 12.

	Group	Week	Diversity
*All Phyla*	GT	0	3.5±0.2
		12	3.4±0.2
	PL	0	3.1±0.3
		12	3.4±0.2
*Bacteroidetes*	GT	0	2.7±0.2
		12	2.5±0.2
	PL	0	2.6±0.2
		12	2.6±0.2
*FAFV*	GT	0	2.2±0.2
		12	2.1±0.2
	PL	0	2.2±0.2
		12	2.0±0.2
*Proteobacteria*	GT	0	2.1±0.2
		12	1.9±0.2
	PL	0	2.1±0.2
		12	1.9±0.2

Green tea (GT, n = 30) and Placebo (PL, n = 28), FAFV: *Firmicutes*, *Actinobacteria*, *Fusobacteria* and *Verrucomicrobia*. Shannon diversity index, Values are means ± standard errors. Data were analyzed by a mixed ANOVA. No significant differences between groups (GT vs. PL), no changes over time (baseline vs. week 12) and no significant interactions were observed between treatment (green tea or placebo) and time (baseline and week 12) for all phyla combined, *Bacteroidetes*, *FAFV* and *Proteobacteria*. All relevant P-values are p>0.05.

**Fig 4:**
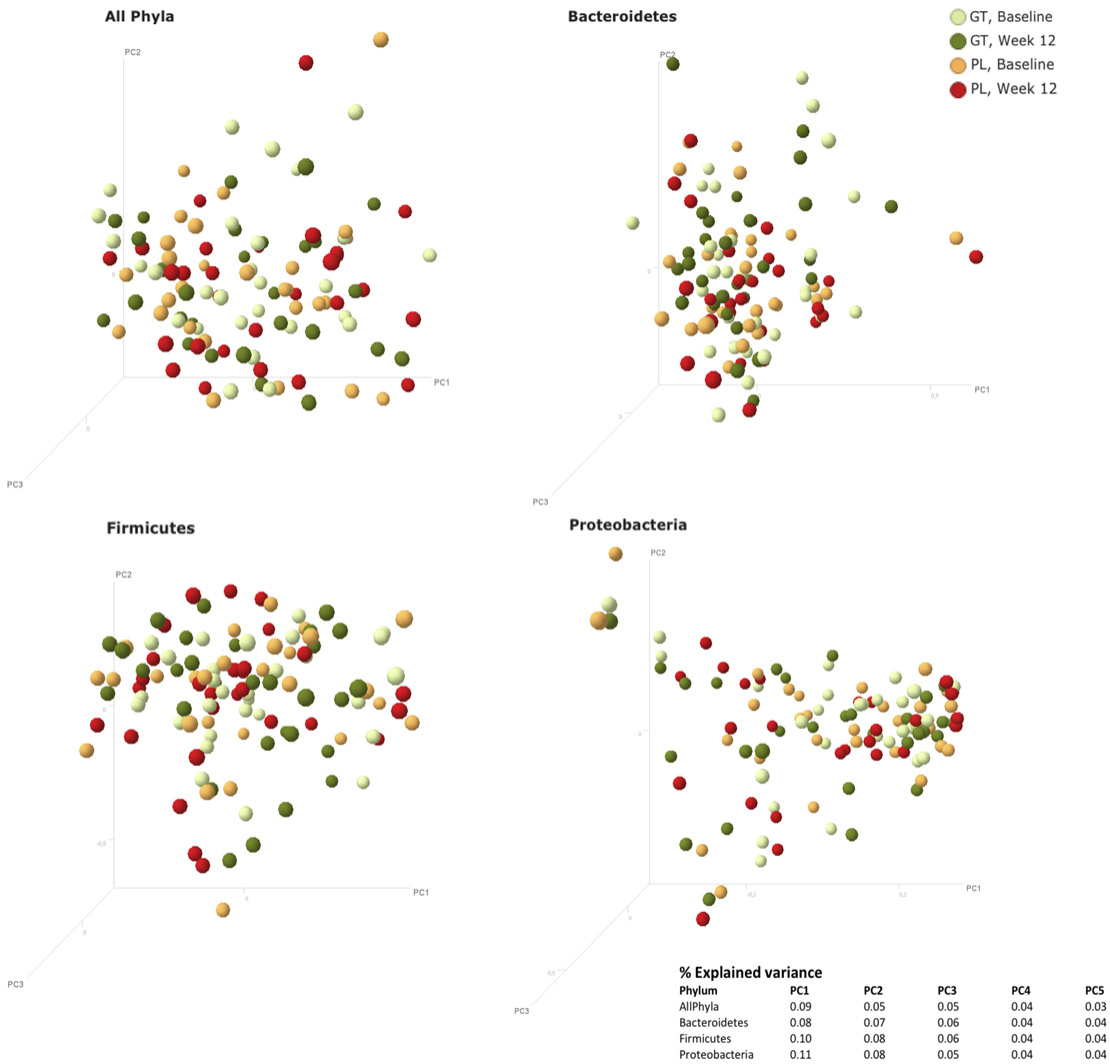
Principal Coordinates Analysis (PCoA) plots of Bray Curtis distances between samples, with the %explained variance by the principle coordinates. PCoA per phylum, Bray Curtis, log2, *n* = 58.

Moreover, clustering according to treatment group was observed neither when visualizing Bray Curtis distances using PCoA graphs for all phyla combined nor for each of the phyla *Bacteroidetes*, *FAFV* and *Proteobacteria* separately (**Fig 4**).

## Discussion

In the present study no significant differences in the fecal bacterial diversity and community structure were observed between baseline and week 12 neither in subjects receiving green tea capsules nor in subjects receiving placebo capsules. Moreover, no significant interactions were observed between treatment (green tea or placebo) and time (baseline and week 12) for the measured variables. Therefore, we conclude that catechin- and caffeine-rich GT supplementation has no long-term effects on composition of the gut microbiota in healthy normal weight and overweight subjects.

We did not observe a significant decrease in body weight and body fat percentage after 12 weeks of GT supplementation [34]. However, another long-term study showed that a mixture of EGCG and caffeine was associated with greater weight maintenance, which was supported by relatively greater thermogenesis and fat oxidation [41]. Therefore, it is likely that GT catechins only have beneficial effects on weight maintenance after weight loss [41], and these effects of catechins on body weight after a diet-induced weight loss may occur via a change in bacterial composition. This may as well be an explanation for the fact that we did not observe a difference in bacterial composition when subjects were in energy balance. In general, the gut microbial composition is quite stable in adults; therefore a change in body weight or extreme switches in dietary patterns may be needed to have a significant change in microbial composition [15, 42]. Furthermore, it is possible that the weight lowering effect of polyphenols only occurs in obese people [43], as supplementation with polyphenols in obese subjects with higher *Firmicutes/Bacteroidetes* ratio was proposed for weight loss [13].

Previous studies did find prebiotic effects of catechins on specific bacterial species. Most of these studies found that catechins may inhibit pathogenic bacteria and may stimulate beneficial bacteria [29–33]. Apart from a study of Jin *et al*, in which an overall tendency for an increase in *Bifidobacteria* was found using qPCR [33], bacteria in the aforementioned studies were cultured, and as 60–80% of the bacterial species cannot be cultured most of the species were uncharacterized [44]. Although, in these studies associations with several individual bacterial species were found, results on total bacterial composition and diversity were lacking. Furthermore, results of previous studies on catechins and gut microbiota are inconsistent in bacterial species that are associated with catechins. Our study is the first study to compare the human gut microbiota community structure and diversity after GT and PL supplementation by means of a comprehensive profiling method.

In the present study catechin- and caffeine-rich GT extract supplements were used. Although a favorable effect of catechin on gut microbiota was expected, the caffeine present may also affect the gut microbiota. However, only few studies investigated the effect of caffeine consumption on gut microbiota. A study in high-fat fed rats showed that caffeine attenuated the increase in *Firmicutes to Bacteroidetes* ratio, which normally occurs after a high-fat diet [45]. In humans no effect of caffeine on the dominant bacteria in the intestines was found [46], these findings are consistent with the results of the current study.

There was no significant difference between normal weight and overweight subject in their response to GT. However, when subjects were divided in two BMI categories (normal weight and overweight), the overall microbial diversity was significantly lower in overweight as compared to normal weight subjects. These findings are in agreement with several previous studies that also indicated a reduced diversity in obese subjects [22, 23]. In a study with 154 subjects (including monozygotic and dizygotic twins) a reduced bacterial diversity was found in obese subjects compared with lean subjects [22]. Main limitation of this study is the number op overweight participants. Although we conducted a study with a rather large sample size, it would have been better if normal weight and overweight subjects were equally divided among participants. Unfortunately we did not manage to find the same number of subjects with a BMI of 18–25 kg/m^2^ as with BMI > 25 kg/m^2^.

This study has shown that long-term catechin- and caffeine-rich GT supplementation had no effect on composition of the gut microbiota as no significant changes over time (baseline vs. week 12) were observed for the measured variables. Moreover, no significant interactions were observed between treatment (green tea or placebo) and time (baseline and week 12). Independently of treatment, we did find a reduced diversity in overweight subjects compared with normal weight subjects. We conclude that green tea supplementation for 12 weeks did not have a significant effect on composition of the gut microbiota.

## Supporting Information

S1 CONSORT Checklist (DOC)

S1 Dataset Raw data on abundance of bacterial groups. (CSV)

S1 Fig Heat map of all profiles sorted by phylum and clustered by the total profile, *n* = 58. Green tea *n* = 30, baseline (light green) and week 12 (dark green); placebo *n* = 28, baseline (orange) and week 12 (red), 2LOG. Red signals represent dominant IS fragment lengths and blue signals represent less prevalent IS fragment lengths. The parentheses below the colored bars were used to display the baseline samples and week 12 samples of the same subject.
(TIFF)

S1 Protocol Study Protocol. (PDF)

S1 Table Mean reported energy and macronutrient intake per day in the green tea and placebo groups. (DOCX)

## Abbreviations

BMI: Body mass index
EGCG: Epigallocatechin-3-gallate
FAFV: *Firmicutes*, *Actinobacteria*, *Fusobacteria* and *Verrucomicrobia*
FMI: Fat mass index
GT: Green tea
IS-PRO: Interspacer profiling
PL: Placebo
WHR: Waist-to-hip ratio
