# Supplementary material for: Long-Term Green Tea Supplementation Does Not Change the Human Gut Microbiota
Source: PLoS One. 2016 Apr 7;11(4):e0153134. doi: 10.1371/journal.pone.0153134 (PMC4824458; doi:10.1371/journal.pone.0153134)
Supplement: S1 Protocol — (PDF) [file pone.0153134.s004.pdf]

C1. Onderzoeksprotocol

RESEARCH PROTOCOL

June 2012

PROTOCOL TITLE

'Long-term effects of green tea on gut flora, fat absorption, body composition and resting energy expenditure'

**Protocol ID**	NL
**Short title**	Long-term effects of green tea
**Version**	2
**Date**	June 2012
**Coordinating investigator**	Prof. dr. M.S. Westerterp-Plantenga<br>Maastricht University, Department of Human Biology, Nutrition and Toxicology Research Institute Maastricht (NUTRIM)<br>P.O. Box 616, 6200 MD Maastricht<br>The Netherlands<br>E-mail: m.westerterp@maastrichtuniversity.nl<br>Phone: +31433881566<br>Fax: +31433670976
**Sponsor**	Maastricht University
**Principal investigators**	Dr. R. Hursel<br>Maastricht University, Department of Human Biology, Nutrition and Toxicology Research Institute Maastricht (NUTRIM)<br>P.O. Box 616, 6200 MD Maastricht<br>The Netherlands<br>E-mail: rick.hursel@maastrichtuniversity.nl<br>Phone: +31433881669<br>Fax: +31433670976
	MSc P.L.H.R. Janssens<br>Maastricht University, Department of Human Biology, Nutrition and Toxicology Research Institute Maastricht (NUTRIM)<br>P.O. Box 616, 6200 MD Maastricht<br>The Netherlands<br>E-mail: plhr.janssens@maastrichtuniversity.nl<br>Phone: +31433884596<br>Fax: +31433670976
**Legal representative**	Nutrition and Toxicology Research Institute Maastricht (NUTRIM)<br>P.O. Box 616, 6200 MD Maastricht<br>The Netherlands<br>E-mail: mh.grispen@maastrichtuniversity.nl<br>Phone: +31433881476<br>Fax: +31433670286
**Independent physician**	Prof. dr. W.H. Lamers,<br>Maastricht University, Department of Anatomy & Embryology<br>Maastricht University Medical Centre+ (MUMC+),<br>P.O. Box 616, 6200 MD Maastricht<br>The Netherlands<br>E-mail: wh.lamers@maastrichtuniversity.nl<br>Phone: +31 43 388 1061
**Laboratory sites**	Maastricht University

PROTOCOL SIGNATURE SHEET

Name	Signature	Date
**Legal representative**<br>NUTRIM<br>Sef Janssen		
**Coordinating Investigator**<br>Prof. dr. M.S. Westerterp-Plantenga		

TABLE OF CONTENTS

1. INTRODUCTION AND RATIONALE 8
2. OBJECTIVES 10
3. STUDY DESIGN 11
3.1. Study design 11
3.2. Intervention 11
3.3. Flow chart 11
4. STUDY POPULATION 12
4.1. Population 12
4.2. Inclusion criteria 12
4.3. Exclusion criteria 12
4.4. Sample size calculation 13
5. TREATMENT OF SUBJECTS 14
5.1. Investigational product/treatment 14
5.2. Use of co-intervention 14
6. METHODS 15
6.1. Study parameters/endpoints 15
6.1.1. Main study parameter/endpoint 15
6.1.2. Secondary study parameters/endpoints 15
6.2. Randomisation, blinding and treatment allocation 15
6.3. Study procedures 15
6.3.1. Body composition 15
6.3.3. REE and substrate oxidation. 16
6.3.4. Physical activity 16
6.3.5 Energy expenditure 17
6.3.6. Energy intake and food choice 17
6.4. Withdrawal of individual subjects 18
6.5. Replacement of individual subjects after withdrawal 18
6.6. Follow-up of subjects withdrawn from treatment 18
7. SAFETY REPORTING 19
7.1. Section 10 WMO event 19
7.2. Adverse and serious adverse events 19
7.3. Follow-up of adverse events 20
8. STATISTICAL ANALYSIS 21
8.1. Descriptive statistics 21
8.2. Univariate analysis 21
9. ETHICAL CONSIDERATIONS 22
9.1. Regulation statement 22
9.2. Recruitment and consent 22
9.3. Benefits and risks assessment, group relatedness 22
9.4. Compensation for injury 23
9.5. Incentives 23
10. ADMINISTRATIVE ASPECTS AND PUBLICATION 24
10.1. Handling and storage of data and documents 24

10.2. Amendments 24
10.3. Annual progress report 24
10.4. End of study report 24
10.5. Public disclosure and publication policy 24
11. REFERENCES 25

LIST OF ABBREVIATIONS AND RELEVANT DEFINITIONS

BMI	Body mass index
CCMO	Central Committee on Research Involving Human Subjects (in Dutch: Centrale Commissie Mensgebonden Onderzoek)
CV	Curriculum Vitae
DIT	Diet induced thermogenesis
EE	Energy expenditure
EGCG	Epigallocatechin gallate
FA	Fatty acids
FFM	Fat free mass
FM	Fat mass
FMI	Fat mass index
IC	Informed Consent
IMP	Investigational Medicinal Product
METC	Medical Research Ethics Committee (MREC) (in Dutch: Medisch Ethische Toetsing Commissie)
PAL	Physical activity
REE	Resting energy expenditure
RQ	Respiratory quotient
(S)AE	(Serious) Adverse Event
SEM	Standard error of the mean
SPSS	Statistical Package for the Social Sciences
TBW	Total body water
TFEQ	Three Factor Eating Questionnaire (TFEQ)
WHR	Waist-to-hip ratio
Wbp	Personal Data Protection Act (in Dutch: Wet bescherming persoonsgevens)
WMO	Medical Research Involving Human Subjects Act (in Dutch: Wet Medisch-wetenschappelijk Onderzoek met Mensen

SUMMARY

Rationale: Green tea might have positive effects for weight control and body composition via several approaches such as a positive effect on the gut flora, a decrease in fat absorption from the intestines and an increase in resting energy expenditure.

Objective: To investigate the long-term effects of green tea on gut flora, fat absorption, resting energy expenditure and body composition.

Study design: The study will be conducted in a randomized, placebo-controlled, double-blind parallel design with four groups consisting of control groups and green tea groups with normal weight subjects and obese subjects. At three time points (baseline, 6 weeks and 12 weeks) faeces are collected for analyzing the gut flora and fat absorption. Furthermore, measurements of resting energy expenditure and body composition will be conducted. Activity will be measured throughout 3 weeks.

Study population: a hundred healthy subjects (50 males and 50 females) with a body mass index (BMI) between 18.5-25 kg/m^2 and ≥30 kg/m^2 and aged between 18-50 years will be included in the study. All subjects will be non-smoking, weight stable, dietary unrestraint, not drinking tea and at most moderate alcohol and caffeine consumers. Subjects will be free of medication except for oral contraceptives use in women.

Intervention: Subjects will receive either green tea or placebo in capsule form after their baseline measurement, which they have to consume daily for a period of twelve weeks.

Main study parameters/endpoints: Gut flora composition, fat absorption, body composition and resting energy expenditure.

Nature and extent of the burden and risks associated with participation:

The study does not include any major risk for the subjects. The collection of faeces will be harmless. Anthropometric and body composition measurements will not be invasive for the subjects. Deuterium dilution has been shown to be a safe method for determining total body water. Furthermore, the registration of oxygen consumption and carbon dioxide production with the ventilated hood will be performed. The air in the hood is continuously regulated.
Administering green tea will not form any health risk. Green tea is a natural product.

Benefit: This study does not have any benefits for the subjects themselves, but will give possible new knowledge for the treatment of obesity.

1. INTRODUCTION AND RATIONALE

Obesity is a result of an energy imbalance that develops when energy intake exceeds energy expenditure. It is associated with an increased risk on developing Type 2 Diabetes, cardiovascular diseases and several cancers (1). Obesity has also been shown to be associated with an increased risk of mortality; overweight and obesity are the fifth leading risk for global deaths (2).

Tea is a widely consumed beverage. Green tea is made from the fresh leaves of *Camillia sinensis L*. and contains polyphenols. The main green tea polyphenols are catechins. The health beneficial effects of green tea have been attributed mainly to these catechins. The most abundant catechin is epigallocatechin gallate (EGCG).

Green tea might have positive effects for weight control and body composition via several approaches such as a positive effect on the gut flora, a decrease in fat absorption from the intestines and an increase in resting energy expenditure.
Green tea appeared to increase the 24-h energy expenditure (EE) and fat oxidation in humans (3). Thereby green tea can induce weight loss and is thought to be useful as an antiobesity compound.

Furthermore, green tea polyphenols might also interfere with fat absorption in the intestine, resulting in decreases in the digestible energy of the diet. A decrease in fat absorption, inferred from increases in faecal fat excretion has been observed in several animal studies (4-6). Raederstorff et al investigated the effect of green tea catechins on lipid metabolism in rats. They showed that EGCG decreased plasma total cholesterol and non-HDL cholesterol concentrations and increased faecal excretion of total lipids and cholesterol (6).

Another animal study also investigated the effect of (EGCG) on obesity and energy metabolism in mice. EGCG attenuated diet-induced body fat accretion in mice and apparently promoted fat oxidation (4).

EGCG has been reported to inhibit the intestinal absorption of lipids and it is suggested that one of the underlying mechanisms by which EGCG affects lipid metabolism is by interfering with the micellar solubilisation of cholesterol in the digestive tract, which then in turn decreased cholesterol absorption (5, 6).

It is also suggested that green tea polyhenols have a positive effect on the gut flora. Trillions of bacteria (microbiota) live in the human gastrointestinal tracts and they all have their own genome. Recent evidence suggests that the trillions of bacteria that normally reside within the human gi-tract affect nutrient acquisition and energy regulation (7). Two groups of beneficial bacteria are dominant in the human gut, the *Bacteroidetes* and the *Firmicutes*. The relative proportion of *Bacteroidetes* to *firmicutes* is decreased in obese people compared to lean people. Obese and lean people have different gut microbiota. This finding raise the posibilty that the gut microbiota has an important role in regulating weight (8)
Green tea is rich in polyphenols. A supplementation with polyphenols with high bioavailability in obese individuals, with higher *Firmicutes/Bacteroidetes* community ratio phenotype, when associated to a probiotic restricted diet, is proposed for weight loss (9).

Green tea might have a positive effect on the gut flora, a decrease in fat absorption from the intestines, an increase in resting energy expenditure and a decrease in the *Firmicutes/Bacteroidetes* ratio. Several animal studies investigated the effect of green tea consumption on fat absorption however it is necessary to investigate whether green tea also lowers the fat absorption and increases the fat excretion in humans. Therefore we hypothesize that green tea consumption has positive effects for weight control via a decrease in fat absorption, an increase in fat excretion and a decrease in the *Firmicutes/Bacteroidetes* ratio.

2. OBJECTIVES

The objective of this study is to investigate the long-term effects of green tea on gut flora, fat absorption, resting energy expenditure and body composition. The primary aim of the present study is to examine whether green tea supplements affect fat metabolism, gut flora and body weight.

This primary objective implies that green tea increases faecal fat and decreases fat absorption, resulting in decrease in digestible energy of the diet. Green tea may inhibit lipase activity. Inhibition of digestive lipase activity could significantly affect dietary lipid absorption and could increase lipid excretion into the faeces. Faecal fat excretion will be measured to determine the lipid absorption (10).
Previous studies have shown comparable effects in animal studies (4-6). This study needs to determine whether green tea also increases faecal fat and lowers the fat absorption in humans and will test the hypothesis that the increase in faecal fat will be larger in the green tea conditions compared to the conditions without green tea.

Furthermore we will investigate whether green tea has positive effects on gut flora and will increase the relative proportion of *Bacteroidetes* to *Firmicutes* and that this change in *Firmicutes/Bacteroidetes* ratio will be larger in obese subjects compared to normal weight subjects. Metabolism of polyphenols by microbiota involves the cleavage of glycosidic linkages. Glycans, which are the product of glycosidic cleavage, are necessary for survival of the intestinal microbiota as a nutrient foundation. *Firmicutes* possess a disproportionately smaller number of glycan-degrading enzymes than *Bacteroidetes*. Furthermore, *Firmicutes* are more repressed than the *Bacteroidetes* by phenolic compounds' antimicrobial properties. The *Bacteroidetes* community prevails following dietary polyphenol intake and its fermentation to phenolic compounds, due to having more glycan-degrading enzymes, so this may thus be a mechanism by which dietary polyphenols exert their weight lowering effect (9).

Finally, we will investigate whether a supplementation with green tea polyphenols is proposed for weight loss via an increase in the resting energy expenditure and fat oxidation.

Four groups consisting of control groups and green tea groups with normal weight subjects and obese subjects will be applied in order to completely answer the research questions:
- Normal weight without green tea (NW/control)
- Obese without green tea (Ob/control)
- Normal weight with green tea (NW/GT)
- Obese with green tea (Ob/GT)

3. STUDY DESIGN

3.1. Study design

The study will be conducted in randomized, placebo-controlled, double-blind parallel design with four randomly sequenced experimental conditions.

3.2. Intervention

Subjects will visit the university three times (baseline, 6 weeks and 12 weeks) and three days before each visit faeces will be collected for analysing the gut flora and fat absorption. They will be asked to travel by public transport or car, in order to avoid physical activity that would increase resting energy expenditure (REE). Subjects will arrive in fasted state at 08:30 h. They will empty their bladder before the test. After resting on a bed for 30 min, the REE and the substrate oxidation of the subjects will be measured for 30 min by means of an open-circuit, ventilated hood system. Subjects will receive either green tea or placebo in capsule form after their baseline measurement, which they have to consume daily for a period of twelve weeks.

All faeces will be collected in pre-weighed plastic containers on the last day before each visit period. Faecal fat excretion will be assessed at baseline and after 6 and 12 weeks. Body weight will also be measured at baseline and after 6 and 12 weeks.

Subjects will be asked not to change their food pattern during this period of twelve weeks. They will be instructed to abstain from tea and dairy products like milk, cheese, pudding and yoghurt and to use less than 100 ml caffeine-containing beverages per day like coffee, cola-type soft drinks and energy drinks.

The subjects will be instructed to maintain their habitual activity level. Physical activity will be determined with the use of a triaxial accelerometer for movement registration (Tracmor; Philips, Eindhoven, Netherlands) during three weeks (baseline, week 6 and week 12).

3.3. Flow chart

	Baseline	6 weeks	12 weeks
Body weight	x	x	x
Fat mass (BodPod0	x	x	x
Collecting feaces	x	x	x
REE	x	x	x
Physical activity	xxx	xxx	xxx
TBW (Deuterium)	x		x
WHR	x		x

4. STUDY POPULATION

4.1. Population

Subjects from the student population will be recruited by advertisements on notice boards at Maastricht University and in local newspapers. Healthy, non-smoking, males and females who are normal weight (Body Mass Index (BMI) 18.5-25 kg/m^2) or obese (BMI ≥30 kg/m^2) and aged between 18-50 yrs will be recruited for an initial screening, whereafter a total of 100 subjects (50 males and 50 females) will be included in the study. During the screening, subjects will undergo anthropometric and body composition measurements, and will complete questionnaires related to health, smoking behaviour, use of medication, alcohol consumption, probiotic consumption, physical activity, eating behaviour, mood and anxiety.

	Age 18-35 years		Age 35-50 years		**Total**
	BMI 18.5-25	BMI > 30	BMI 18.5-25	BMI > 30	
Male	12	12	12	12	48
Female	12	12	12	12	48
**Total**	24	24	24	24	**96**

4.2. Inclusion criteria

A total of 100 subjects (50 males and 50 females) with a BMI between 18.5-25 kg/m^2 and ≥30 kg/m^2 and aged between 18-50 years will be included in the study. All subjects will be non-smoking, healthy, weight stable, dietary unrestraint, not using a more than moderate amount of alcohol (<10 consumptions/wk) or more than 100 mg caffeine per day, not drinking tea, not using probiotics, being weight stable (weight change < 3kg during the last 6 months), dietary unrestraint and not using antibiotics during the last 6 months. The Dutch translation of the Three Factor Eating Questionnaire (TFEQ) will be used to determine eating behaviour (11). Non-restrained eaters (<9 times factor 1), these are persons who are not consciously occupied with food and who are caloric restricted, will be selected. Subjects will be free of medication except for oral contraceptives use in women.

4.3. Exclusion criteria

Subjects will be excluded if they are not healthy, smoking, using a more than moderate amount of alcohol (>10 consumptions/wk), using more than 100 mg caffeine per day, drinking tea, using probiotics, not being weight stable, dietary restraint, using medication or supplements except for oral contraceptives in women, using antibiotics or if they do not meet the criteria for BMI and age. Pregnant and lactating women, and subjects with allergies for the used food items will also be excluded from participation.

4.4. Sample size calculation

The primary endpoint of this study is the change in gut flora and fat absorption.

For the sample size calculation the following formula was used:

$N = 2 + (Z_{1\text{-}\beta} + Z_{1\text{-}\alpha / 2})^2 \;\text{x}\; \sigma^2 / (\mu_{GT} - \mu_{CONTROL})^2$

Subject population size was calculated using G*Power 3.1.2 where α was 0.05, β was 0.95 using group mean body weight changes from past papers (12) to calculate the effect size, the population was finalized as 45 per group and an additional 5 (drop-out of 10%) will be included in the population size for any unforeseeable circumstances which may lead to subjects dropping out.

The calculated sample size will be:

Means:	u1 = 72.5	u2 = 71.4
Standard deviation	SD1= 1.4	SD2= 1.7

Confidence interval is α= 0.05, for β=0.95

$$N1 = N2 = 2 + (Z(1\text{-}\beta) + Z\,(1\text{-}\alpha/2))^2 * \frac{(\sigma 1^2 + \sigma 2^2)}{(\mu 1 - \mu 2)^2}$$

45 people per condition (two conditions; green tea and placebo).

Taking a drop-out of 10% into account, 50 subjects will be included per condition and herewith 100 subjects will be included in the experiment.

5. TREATMENT OF SUBJECTS

5.1. Investigational product/treatment

Subjects will receive either green tea or placebo in capsule form after their baseline measurement, which they have to consume daily for a period of twelve weeks. Subjects will visit the university three times (baseline, 6 weeks and 12 weeks) and three days before each visit faeces will be collected for analysing the gut flora and fat absorption. They will be asked to travel by public transport or car, in order to avoid physical activity that would increase REE. Subjects will arrive in fasted state at 08:30 h. They will empty their bladder before the test. After resting on a bed for 30 min, the REE and the substrate oxidation of the subjects will be measured for 30 min by means of an open-circuit, ventilated hood system.

Subjects will receive either green tea (250 mg/capsule [> 62.5 mg EGCG + > 31.3 mg caffeine], 3 capsules with each breakfast, lunch and dinner) or placebo (soy oil; 757 mg/capsule, 3 capsules with each breakfast, lunch and dinner) in capsule form after their baseline measurement, which they have to consume daily for a period of twelve weeks. The product information for the green tea and the placebo capsules can be found in the attachments. When specifying the placebo capsules the green tea extract is replaced with microcrystalline cellulose (270.5 mg per capsule in the green tea capsules vs 515.5 mg in the placebo capsules). Microcrystalline cellulose is refined wood pulp, and often used in vitamine supplement.

5.2. Use of co-intervention

Subjects will be asked not to change their food pattern during the period of twelve weeks. They will be instructed to abstain from dairy products (like milk, cheese, pudding and yoghurt) to use less than 100 ml caffeine-containing beverages per day and to avoid probiotics.

6. METHODS

6.1. Study parameters/endpoints

6.1.1. Main study parameter/endpoint

The primary endpoint of this study is the change in gut flora; ratio firmicutes/bacteroidetes, changes in total faecal fat and change in body weight.

Green tea may inhibit lipase activity, which is essential for lipid absorption. Faecal fat excretion will be measured to determine the lipid absorption.

6.1.2. Secondary study parameters/endpoints

- Resting energy expenditure (REE)
- Respiratory quotient (RQ)
- Three frequency eating questionnaire (TFEQ)
- Body composition: BMI, body fat percentage, fat mass index (FMI)
- Body fat distribution: waist circumference, waist-to-hip ratio (WHR)

6.2. Randomisation, blinding and treatment allocation

The study will be conducted in a randomized, placebo-controlled, double-blind parallel design with four randomly sequenced experimental conditions. Randomisation will take place using a computerized randomization program (‘ Randomizer’ macro in Excel).

6.3. Study procedures

6.3.1. Body composition

Body weight will be measured using a digital balance and height by a wall-mounted stadiometer. BMI will be calculated as body weight (kg) divided by height (m) squared. The deuterium dilution method according to the Maastricht protocol will be used to determine total body water (TBW) (13). The subjects will be asked to collect a urine sample in the evening just before drinking a deuterium-enriched water solution. After ingestion of this solution, the subject will go to bed and no additional consumption is allowed for that period. Ten hours after drinking the water solution, another urine sample will be collected. The dilution of the deuterium isotope is a measure of the TBW of the subject. Fat mass (FM) will be calculated as body weight minus TBW divided by the hydration factor 0.73 (14). Additionally, fat mass will be determined by Bodpod measurements. Fat mass index (FMI) will be calculated by fat mass (kg) divided by height (m) squared. BMI, FM (%) and FMI will be used to define body composition. Waist and hip circumference will be determined in standing position by a tape measure. Waist circumference will be measured at the smallest circumference between rib cage and iliac crest, and hip circumference at the level of the spina iliaca anterior superior.

Accordingly, WHR will be calculated by dividing waist by hip circumference. Both waist circumference and waist-to-hip ratio will be used to define different patterns of body fat distribution.

6.3.2. Attitude toward eating.

To determine whether attitude toward food intake changed during the experiment, the three-factor eating questionnaire was used. Factor 1 indicates cognitive dietary restraint, factor 2 indicates disinhibition of eating, and factor 3 indicates general hunger feelings.

6.3.3. REE and substrate oxidation.

REE and substrate oxidation will be measured by means of an open-circuit-ventilated hood system. After 30 min of resting, to make sure that the subjects are rested, REE will be measured in the morning with subjects in a fasted state while lying supine for 30 min. Gas analyses will be performed by a paramagnetic oxygen analyzer (OmniCal type 1155B; Servomex, Crowborough Sussex, United Kingdom) and an infrared carbon dioxide analyzer (OmniCal type 1520/1507; Servomex). Calculation of REE is based on Weir's formula (15). Respiratory quotient will be calculated as carbon dioxide produced/oxygen consumed. Alternately, before or after each REE measurement, the function of the ventilated-hood system will be checked with methanol burning during 20 min. The methanol burner will be set to burn 0–2 g/min, which is equivalent to the production of 150 mL CO2/ min and the consumption of 225 mL O2/min. Because an error percentage depends on burn rate, the expression of the error limit in absolute mL/min is preferred; hence, the limit values of 7–5 mL CO2 and 11 mL O2/min, respectively (5% of 150 mL CO2 and 5% of 225 mL O2).

6.3.4. Physical activity

The subjects will be instructed to maintain their habitual activity level. Physical activity will be determined with the use of a triaxial accelerometer for movement registration (Tracmor; Philips, Eindhoven, Netherlands) during three weeks (baseline, week 6 and week 12). The Tracmor is a small device (7 × 2 × 0.8 cm; 30 g), which measures accelerations in the anteroposterior, mediolateral, and vertical directions of the trunk (16). Subjects will wear the same type of accelerometer during waking hours in a belt at the back of the waist, during the three weeks.

PAL (physical activity level) will be calculated with the following equations:

TEE = -1.259 + (1.552 x REE) + (0.076 x counts/min)

PAL = TEE/REE

in which TEE (total energy expenditure) and REE are measured in MJ/d.

6.3.5 Energy expenditure

TEE will be calculated as indicated above.

6.3.6. Energy intake and food choice

Subjects will be asked not to change their food pattern during the period of twelve weeks. However, they will be instructed to abstain from caffeine-rich products like tea, coffee, cola-type soft drinks and energy drinks and not to use dairy products (like milk, cheese, pudding and yoghurt) because consumption of milk-protein inhibits the effect of green tea on diet induced thermogenesis (DIT) (17), To determine whether subjects did not change their food pattern, except as instructed, during the period of twelve weeks they will be asked to fill in a food frequency questionnaire at each visit. During the last four days before the first visit subjects will be asked to maintain their food pattern and to record all the food and drinks they consume. Then, during the last four days before the next visits subjects will be asked to eat and drink the same as on the days four days before the first visit.

6.3.7 Faecal energy, faecal fat and faecal FA composition

All feaces excreted will be collected in preweighed containers during the last three days before each visit. Faeces will be collected during three days according to the standard operating procedure of clinical chemistry. Furthermore, reference values are based on 3x24h faeces. Before analysis, the faecal samples will be freeze-dried and homogenized. For each subject, samples from the same period will be pooled. Faecal energy will be obtained using a bomb calorimeter (Ika-calorimeter system C4000; Heitersheim, Germany). Total faecal fat will be analyzed after the method of Bligh and Dyer (18) with modifications. Before fat extraction, the faecal samples will be acid hydrolyzed with 3 N HCl at 80°C for 1h. During extraction, a known quantity of 17:0 (Sigma H-4515) will be added to all samples as an internal standard for the FA analysis. The faecal FA composition will be determined as described below. The extracted fat will be saponified with 0.8ml methanolic sodium hydroxide (0.5M) by heating at 100°C for 1h. The FAs will be hydrolyzed by the addition of 1ml of boron triflouride/methanol complex (20%) and methylated by heating at 100°C for 45min. The methyl esters will be extracted by 2ml heptane mixed with 4ml of saturated sodium chloride solution. Finally, the heptane fase will be transferred to vials. The FA methyl esters will be analyzed by gas chromatography (HP 6890series GC system; Agilent Technologies, Palo Alto, CA, USA). The gas chromatograph will be equipped with an automatic on-column injector (HP 7673) (split ratio 4.325:1), a capillary column of 30□m × 320μm inner diameter,

0.25μm film thickness (Omegawax; Supelco 4-293-415) and a flame ionization detector. Helium will be used as the carrier gas. The oven temperature will be set at 86°C, 4min isothermal, 10°C min−1 to 210°C, 15min isothermal. The detector will be set at 250°C. Chromatograms will be recorded with a data system integrator (HP Chemstation; Hewlett-Packard, Palo Alto, CA, USA). Identifications of peaks will be made by comparison of the retention times with those of a standard (mix of pure FAs from Sigma-Aldrich, Broendby, Denmark and Merck & Co Inc., NJ, USA) run under identical conditions. The quantity of FAs in each sample will be calculated from the known quantity of internal standard by comparing the area under the curve of each FA methyl ester with that of 17:0 methyl ester. Conjugated and free BAs in faecal samples will be quantified by reversed-phase high-performance liquid chromatography with pulsed amperometric detection as outlined by Dekker et al. (19). The freeze-dried samples will be mixed using a Shaker VXR vibrax (IKA-Werke, Staufen, Germany) at 1500 r.p.m. for 30 s and subsequently centrifuged at 5000 g for 10 min. The supernatant will be passed through a nylon syringe filter membrane (Cameo 17N-DDR02T17NB) before injection onto the high-performance liquid chromatography. The chromatographic conditions that will be used are described by Knarreborg (20).

6.4. Withdrawal of individual subjects

Subjects can leave the study at any time for any reason if they wish to do so without any consequences. The investigator can decide to withdraw a subject from the study for urgent medical reasons.

6.5. Replacement of individual subjects after withdrawal

New subjects will replace subjects that are withdrawed from the study.

6.6. Follow-up of subjects withdrawn from treatment

Subjects withdrawn from treatment will not be followed-up after eventually handing over responsibilities to medical personnel.

7. SAFETY REPORTING

7.1. Section 10 WMO event

In accordance to section 10, subsection 1, of the WMO, the investigator will inform the subjects and the reviewing accredited METC if anything occurs, on the basis of which it appears that the disadvantages of participation may be significantly greater than was foreseen in the research proposal. The study will be suspended pending further review by the accredited METC, except insofar as suspension would jeopardise the subjects' health. The investigator will take care that all subjects are kept informed.

7.2. Adverse and serious adverse events

Adverse events are defined as any undesirable experience occurring to a subject during the study, whether or not considered related to the experimental intervention. All adverse events reported spontaneously by the subject or observed by the investigator or his staff will be recorded.

A serious adverse event (SAE) is any untoward medical occurrence or effect that at any dose:

- results in death;
- is life threatening (at the time of the event);
- requires hospitalisation or prolongation of existing inpatients' hospitalisation;
- results in persistent or significant disability or incapacity;
- is a congenital anomaly or birth defect;
- is a new event of the trial likely to affect the safety of the subjects, such as an unexpected outcome of an adverse reaction, lack of efficacy of an (investigational medicinal product) IMP used for the treatment of a life threatening disease, major safety finding from a newly completed animal study, etc.

All SAEs will be reported through the web portal *ToetsingOnline* to the accredited METC that approved the protocol, within 15 days after the sponsor has first knowledge of the serious adverse reactions.

SAEs that result in death or are life threatening should be reported expedited. The expedited reporting will occur not later than 7 days after the responsible investigator has first knowledge of the adverse reaction. This is for a preliminary report with another 8 days for completion of the report.

7.3. Follow-up of adverse events

All adverse events will be followed until they have abated, or until a stable situation has been reached. Depending on the event, follow up may require additional tests or medical procedures as indicated, and/or referral to the general physician or a medical specialist.

8. STATISTICAL ANALYSIS

8.1. Descriptive statistics

Data will be presented as means ± standard error of the mean (SEM).

The four groups will be stratified according to the number of males and females, BMI, age and the differences between these groups will be checked with the use of a factorial analysis of variance (ANOVA). P-values for these for these differences were, respectively, >0.9 for sex distribution and BMI, >0.8 for age, >0.5 for weight loss.

8.2. Univariate analysis

The Statistical Package for the Social Sciences (SPSS) will be used to perform univariate analyses. ANOVA will be performed to determine possible differences in gut flora, fat absorption, REE, activity and body composition between the four groups, with time (baseline, 6 weeks, 12 weeks) as the within-subject factor and group (normal weight-GT, normal weight-control, obese-GT, obese-control) as the between-subject factor. Post hoc, a Scheffe's *F* test will be applied. Statistical significance will be set at p<.05.

9. ETHICAL CONSIDERATIONS

9.1. Regulation statement

The study will be conducted according to the principles of the Declaration of Helsinki (9th version, October 2008, Seoul) and in accordance with the Dutch version of the Medical Research Involving Human Subjects Act (in Dutch: Wet Medisch-wetenschappelijk Onderzoek met mensen; WMO).

9.2. Recruitment and consent

Subjects from the student population will be recruited by advertisements in local newspapers and on notice boards at the Maastricht University. They will be informed about the study by reading the provided written ‘subject information’. The researchers will orally check their comprehensiveness. An independent person who’s well informed about the study could eventually provide additional information, and subjects will be referred to the ‘Centrale Commissie Mensgebonden Onderzoek’ (CCMO)-brochure ‘General information for research participants’ that contains general information about medical-scientific research. After being well-informed subjects will get one week before they have to decide to participate in the study. All subjects will confirm their approval for participation by signing an informed consent (IC) form.

9.3. Benefits and risks assessment, group relatedness

This study does not have any benefits for the subjects themselves, but will give possible new knowledge for treatment of obesity. Besides, the study does not include any major risk for the subjects. The collection of faeces will be harmless. Anthropometric and body composition measurements, performed within several minutes during the screening, will not be invasive for the subjects. Deuterium dilution has been shown to be a safe method for determining total body water. Subjects have to consume an amount of about 150 mL of water in which a small amount of deuterium is diluted. The taste of the solution is comparable to normal water (21). Furthermore, the registration of oxygen consumption and carbon dioxide production with the ventilated hood will be performed during an unconscious process. The air in the hood is continuously regulated. Administering green tea will not form any health risk. Green tea is a natural product, which is safe in the given dose that will not exceed the maximum recommended daily dose.

The completion of screening questionnaires will take about 30 minutes.

Accidential findings during the screening related to health status will be communicated to the concerned subjects, whereafter they can individually undertake further steps.

Green tea and placebo capsules will be processed, handled, packaged and transported by Taiyo Kagaku Co., Ltd., as described in the certificate of analysis. After arrival at the University the product will be stored in a locked food depot in the research kitchen. Green tea and placebo capsules will be stored for a maximum period of one year, which corresponds to the maximum duration of the study and will not exceed the expiration date of the product. A certified dietician controls the storage of the products.
Further information can be found in the guideline 'MEC azM/UM for preparation, delivery and administration of products for human usage in clinical studies'.

9.4. Compensation for injury
The investigator has a liability insurance, which is in accordance with article 7, subsection 6 of the WMO.

The sponsor also has an insurance which, is in accordance with the legal requirements in the Netherlands (Article 7 WMO and the Measure regarding Compulsory Insurance for Clinical Research in Humans of 23th June 2003). This insurance provides cover for damage to research subjects through injury or death caused by the study.

€ 450.000,-- (i.e. four hundred and fifty thousand Euro) for death or injury for each subject who participates in the Research;

€ 3.500.000,-- (i.e. three million five hundred thousand Euro) for death or injury for all subjects who participate in the Research;

€ 5.000.000,-- (i.e. five million Euro) for the total damage incurred by the organisation for all damage disclosed by scientific research for the Sponsor as 'verrichter' in the meaning of said Act in each year of insurance coverage.

The insurance applies to the damage that becomes apparent during the study or within 4 years after the end of the study.

9.5. Incentives
After completion of the study subjects will receive a financial compensation of 100 euro.
Possible travel expenses will be declared.

10. ADMINISTRATIVE ASPECTS AND PUBLICATION

10.1. Handling and storage of data and documents

Subjects' personal information will be confidentially handled during the study, according to the Dutch Personal Data Protection Act (Wet bescherming persoonsgegevens; Wbp). Results will be linked to the intervention and not to individual subjects. Subject numbers (e.g. number 1) in combination with treatment codes (NW/control, Ob/control, NW/GT, Ob/GT) will be used throughout the study. Only the researcher is familiar with subject information, so privacy of personal information is guaranteed. If desirable, subjects will be informed about their personal study results at the end of the study. Collected body material (urine and faeces) will be destroyed after publication of the results, body material will be kept for a maximum period of 15 years. Personal data will be kept for a period of 15 year, which is the legal time to keep medical data. Hereafter the data will be destroyed.

10.2. Amendments

Amendments are changes made to the research after a favourable opinion by the accredited METC has been given. All amendments will be notified to the METC that gave a favourable opinion.

10.3. Annual progress report

The investigator will submit a summary of the progress of the trial to the accredited METC once a year. Information will be provided on the date of inclusion of the first subject, numbers of subjects included and numbers of subjects that have completed the trial, serious adverse events/ serious adverse reactions, other problems, and amendments.

10.4. End of study report

The investigator will notify the accredited METC of the end of the study within a period of 8 weeks. The end of the study is defined as the last patient's last visit.
In case the study is ended prematurely, the investigator will notify the accredited (Medisch Ethische Toetsing Commissie) METC, including the reasons for the premature termination.
Within one year after the end of the study, the investigator will submit a final study report with the results of the study, including any publications/abstracts of the study, to the accredited METC.

10.5. Public disclosure and publication policy

The 'CCMO Statement on publication policy' will be followed to publish research results after the end of the study.

11. REFERENCES

1. Haslam DW, James WP. Obesity. Lancet. 2005 Oct 1;366(9492):1197-209.
2. WHO. Obesity and overweight. 2011; Available from: http://www.who.int/mediacentre/factsheets/fs311/en/.
3. Dulloo AG, Duret C, Rohrer D, Girardier L, Mensi N, Fathi M, et al. Efficacy of a green tea extract rich in catechin polyphenols and caffeine in increasing 24-h energy expenditure and fat oxidation in humans. Am J Clin Nutr. 1999 Dec;70(6):1040-5.
4. Klaus S, Pultz S, Thone-Reineke C, Wolfram S. Epigallocatechin gallate attenuates diet-induced obesity in mice by decreasing energy absorption and increasing fat oxidation. International Journal of Obesity. 2005 Jun;29(6):615-23.
5. Wang S, Noh SK, Koo SI. Epigallocatechin gallate and caffeine differentially inhibit the intestinal absorption of cholesterol and fat in ovariectomized rats. J Nutr. 2006 Nov;136(11):2791-6.
6. Raederstorff DG, Schlachter MF, Elste V, Weber P. Effect of EGCG on lipid absorption and plasma lipid levels in rats. Journal of Nutritional Biochemistry. 2003 Jun;14(6):326-32.
7. Bajzer M, Seeley, R.J. Obesity and gut flora. nature. 2006;444(21/28):1009-10.
8. Ley RE, Turnbaugh, P.J. Human gut microbes associated with obesity Nature. 2006;444(21):1022-3.
9. Rastmanesh R. High polyphenol, low probiotic diet for weight loss because of intestinal microbiota interaction. Chem Biol Interact. Jan 15;189(1-2):1-8.
10. Hsu TF, Kusumoto A, Abe K, Hosoda K, Kiso Y, Wang MF, et al. Polyphenol-enriched oolong tea increases fecal lipid excretion. Eur J Clin Nutr. 2006 Nov;60(11):1330-6.
11. Stunkard AJ, Messick S. The three-factor eating questionnaire to measure dietary restraint, disinhibition and hunger. J Psychosom Res. 1985;29(1):71-83.
12. Nagao T, Komine Y, Soga S, Meguro S, Hase T, Tanaka Y, et al. Ingestion of a tea rich in catechins leads to a reduction in body fat and malondialdehyde-modified LDL in men. Am J Clin Nutr. 2005 Jan;81(1):122-9.
13. Schoeller DA, van Santen E, Peterson DW, Dietz W, Jaspan J, Klein PD. Total body water measurement in humans with 18O and 2H labeled water. Am J Clin Nutr. 1980 Dec;33(12):2686-93.
14. Westerterp KR, Wouters L, Van Marken Lichtenbelt WD. The Maastricht protocol for the measurement of body composition and energy expenditure with labeled water. Obes Res. 1995;3S1:49-57.
15. Weir JB. New methods for calculating metabolic rate with special reference to protein metabolism. J Physiol. 1949 Aug;109(1-2):1-9.
16. Goris AH, Meijer EP, Kester A, Westerterp KR. Use of a triaxial accelerometer to validate reported food intakes. Am J Clin Nutr. 2001 Mar;73(3):549-53.

17. Hursel R, Westerterp-Plantenga MS. Green tea catechin plus caffeine supplementation to a high-protein diet has no additional effect on body weight maintenance after weight loss. Am J Clin Nutr. 2009 Mar;89(3):822-30.
18. Bligh EG, Dyer WJ. A rapid method of total lipid extraction and purification. Can J Biochem Physiol. 1959 Aug;37(8):911-7.
19. Dekker R, Vandermeer R, Olieman C. Sensitive Pulsed Amperometric Detection of Free and Conjugated Bile-Acids in Combination with Gradient Reversed-Phase Hplc. Chromatographia. 1991 Jun;31(11-12):549-53.
20. Knarreborg A. *The Impact of Microbial Deconjugation of Bile Salts on Fat Digestion in Broiler Chickens.* : The Royal Veterinary and Agricultural University: Frederiksberg Denmark; 2002.
21. van Marken Lichtenbelt WD, Westerterp KR, Wouters L. Deuterium dilution as a method for determining total body water: effect of test protocol and sampling time. The British journal of nutrition. 1994 Oct;72(4):491-7.
