# Supplementary material for: Long-Term Green Tea Supplementation Does Not Change the Human Gut Microbiota
Source: PLoS One. 2016 Apr 7;11(4):e0153134. doi: 10.1371/journal.pone.0153134 (PMC4824458; doi:10.1371/journal.pone.0153134)
Supplement: S1 Table — (DOCX) [file pone.0153134.s005.docx]

S1 Table. Mean reported energy and macronutrient intake per day in the green tea and placebo groups.

|  | Green tea (*n*=20) | Placebo (*n*=21) |
| --- | --- | --- |
| Energy (MJ) | 7.9±2.0 | 8.2±1.8 |
| *Fat* |  |  |
| Fat (g) | 76.4±25.7 | 78.6±26.4 |
| Fat (En%) | 35.8±4.6 | 35.4±6.5 |
| *Protein* |  |  |
| Protein (g) | 72.2±18.1 | 70.7±20.1 |
| Protein (En%) | 15.4±2.2 | 14.5±2.9 |
| *Carbohydrate* |  |  |
| Carbohydrate (g) | 215.0±52.2 | 224.6±46.9 |
| Carbohydrate (En%) | 45.6±3.8 | 46.4±6.4 |
| *Alcohol* |  |  |
| Alcohol (mg) | 1.9±3.7 | 3.2±5.7 |
| Alcohol (En%) | 0.6±1.1 | 0.9±1.6 |
| Dietary fibre, g | 17.4±5.3 | 18.7±6.5 |
| Minerals |  |  |
| Sodium, g | 2.9±0.8 | 3.1±1.0 |
| Potassium, g | 2.5±0.6 | 2.6±0.7 |
| Calcium, g | 0.7±0.2 | 0.7±0.3 |
| Magnesium, g | 0.2±0.1 | 0.3±0.1 |
| Iron, mg | 9.0±3.5 | 9.0±3.2 |
| Selenium, μg | 40.3±13.7 | 40.8±16.6 |
| Zinc, mg | 8.1±2.4 | 8.1±3.7 |
| Vitamins |  |  |
| Vitamin A, mg | 0.6±0.3 | 0.8±0.9 |
| Vitamin B1, mg | 1.0±0.4 | 0.8±0.3 |
| Vitamin B2, mg | 1.2±0.5 | 1.2±0.6 |
| Vitamin B3, mg | 14.7±6.0 | 14.6±6.7 |
| Vitamin B6, mg | 1.4±0.8 | 1.3±0.6 |
| Folic acid, mg | 0.2±0.1 | 0.2±0.1 |
| Vitamin B12, μg | 3.3±1.6 | 4.2±4.5 |
| Vitamin C, mg | 72.6±35.5 | 67±34.7 |
| Vitamin D, μg | 2.2±1.0 | 2.3±1.6 |
| Vitamin E, mg | 11.2±5.0 | 9.7±3.9 |

Energy and macronutrient intake was recorded during four consecutive days before baseline and week 12. Data are means ± standard deviations. Data were analyzed by repeated-measures ANOVA. No significant differences between groups (green tea vs. placebo) were observed.
